# The complete chloroplast genome of *Epimedium pudingense* (Berberidaceae), a narrowly distributed plant species in China

**DOI:** 10.1080/23802359.2020.1781560

**Published:** 2020-07-02

**Authors:** Yixin Zhang, Xiang Liu, Yu Yao, Yanjiao Luo, Qianru Yang, Cheng Zhang, Xu Chaoqun, Fengmei Suo, Guoan Shen, Baolin Guo

**Affiliations:** a Institute of Medicinal Plant Development, Chinese Academy of Medical Science, Peking Union Medical College, Beijing, China; b Chongqing Academy of Chinese Materia Medica, Chongqing, China; c College-of-Pharmacy, Shanxi Medical University, Taiyuan, China

**Keywords:** Chloroplast genome, *Epimedium pudingense*, Berberidaceae

## Abstract

*Epimedium* L. is the largest herbaceous genus of Berberidaceae which comprises more than 60 species. *Epimedium pudingense* is a rare plant species only narrowly inhabited in the Puding County in the Guizhou Province of China. Here, we first report the complete chloroplast genome of *E. pudingense* assembled from Illumina short-read sequencing data. The chloroplast genome of *E. pudingense* was 157,325 bp in length, with a total GC content of 36.11%. A total of 112 unique genes were identified, among which are 78 protein-coding genes, 30 tRNA genes and four rRNA genes. The phylogenetic analysis revealed that *E. pudingense* closely related to *E. elachyphyllum*. Our study will provide useful fundamental data for further phylogenetic and evolutionary studies of the *Epimedium* genus.

*Epimedium* L., the largest herbaceous genus of Berberidaceae, comprises more than 60 species discontinuously distributed in the temperate zone of Eurasia. (Zhang et al. 2014). China is the distribution and diversity center for possession of more than 50 species (mainly concentrate in the Hubei, Sichuan, and Guizhou Provinces). Due to their nourishing effect on kidney, muscles and bones, *Epimedium* plants have been used as traditional Chinese medicine “herba epimedii” for more than 2000 years. Modern pharmacological studies showed that herba epimedii has wide-reaching activities, including regulating bone remodeling, curing cardiovascular diseases, anti-tumor benefits and so on (Fan and Quan 2012).

*Epimedium* is one of the most taxonomically and phylogenetically intractable genus, since interspecific hybridization and gene introgression complicated the interspecific relationship. In recent years, the complete chloroplast genome has shown many great advantages in plant phylogenetic research, such as mass storage of information, relatively conserved gene content and genome structure, moderate nucleotide substitution rate and so on (Zhang and Li 2011). Considering how large and complex this genus is, there is still more species need to be sequenced and assembled to clarify the phylogenetic question.

*Epimedium pudingense* is a rare species only distributed in the Puding County of Guizhou Province in China, and it is used as herba epimedii by local people. Furthermore, *E. pudingense* is a medicinal *Epimedium* germplasm resource peculiar to the Guizhou Province in China (He et al. 2011). In this study, we present the first complete chloroplast genome of *E. pudingense*. The results will provide more useful information for phylogenetic and evolutionary research of the genus *Epimedium*.

For this study, the *E. pudingense* was sampled from the Puding County of Guizhou province (China; 29°19′ N, 105°44′E). A voucher specimen (Guo0348) was deposited at the Herbarium of the Institute of Medicinal Plant (IMPLAD), Beijing, China. Total genomic DNA was extracted from the fresh leaves of *E. pudingense* using the modified CTAB method (Doyle and Doyle 1987). The high-quality DNA was sheared to the size of 300 bp for the shotgun library construction. Genome sequencing was performed using Illumina Novaseq PE150 platform (Illumina Inc, San Diego), and 150 bp paired-end reads were generated. We assembled the chloroplast genome using the program GetOrganelle version 1.6.2 (Jin et al. 2018) with *E. acuminatum* chloroplast genome (GenBank accession number: NC_029941) as a reference. The annotation of chloroplast genome was conducted through the online program CPGAVAS 2 (Shi et al. 2019), and assisted with careful manual correction. The annotated genomic sequence was registered into GenBank with an accession number (MT506232).

The complete chloroplast genome of *E. pudingense* was 157,325 bp in length, consisting of a large single copy region (LSC, 88,635 bp), a small single copy region (SSC, 17,050 bp), and two inverted repeat regions (IRa and IRb, 25,820 bp). The total GC content was 38.76%, with IR regions (43.17%) higher than that in LSC (37.35%) and SSC regions (32.77%). A total of 112 unique genes were identified from the chloroplast genome of *E. pudingense*, among which are 78 protein-coding genes, 30 tRNA genes and four rRNA genes. The intron-exon structure analysis indicated that 18 genes have introns, among which *petB*, *petD*, *rpl16*, *rpl2*, *rpoC1*, *rps16*, *trnA*-*UGC*, *trnG*-*UCC*, *trnI*-*GAU*, *trnK*-*UUU*, *trnL*-*UAA*, *trnV*-*UAC*, *atpF*, *ndhA*, and *ndhB* have one intron, while *ycf3*, *rps12*, and *clpP* have two introns.

To identify the phylogenetic position of *E. pudingense*, we downloaded the complete chloroplast genome sequences of 16 species from the NCBI GenBank database. MAFFT v7 (Katoh et al. 2017) was used for sequence alignment, and then a maximum likelihood tree was constructed by using RAxML v8.2.10 (Stamatakis 2014) with *Clematis terniflora* as the outgroup (Figure 1). The phylogenetic analysis revealed that *E. pudingense* closely clustered with *E. elachyphyllum*. Our study will provide essential data for future research on the phylogenetic and evolutionary relationship in *Epimedium* genus.

**Figure 1.**
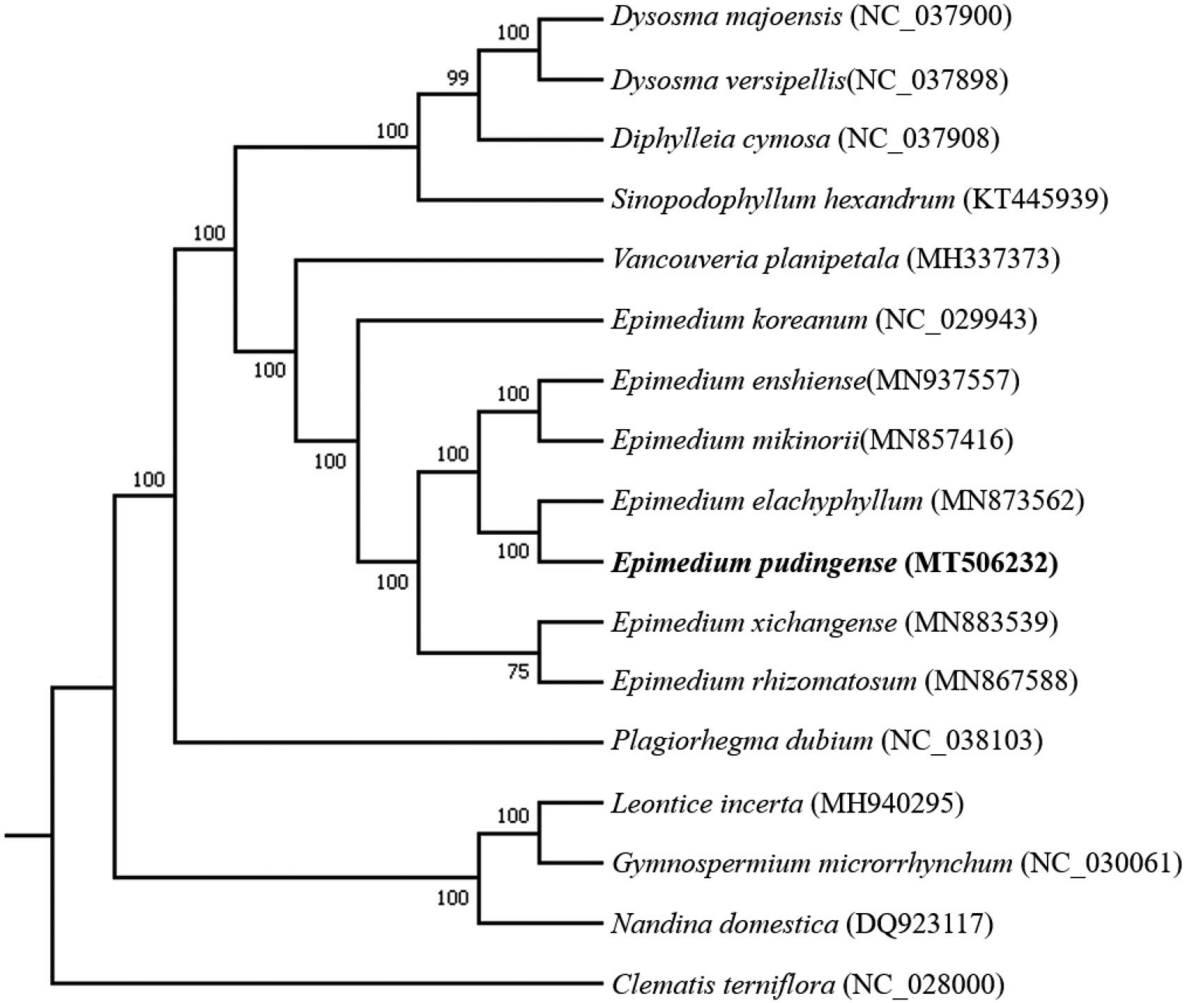
Maximum likelihood (ML) phylogenetic tree based on complete chloroplast genomes of 17 species, with *Clematis terniflora* as outgroup. Numbers at nodes represent bootstrap values.

## Data Availability

The data that support the findings of this study are openly available in NCBI Sequence Read Archive at https://trace.ncbi.nlm.nih.gov/Traces/sra/?study=SRP265549, reference number: SRP265549.
